# Supplementary material for: Audit of antibiotic utilization patterns and practice for common eye infections at the ambulatory clinic of a teaching hospital in Ghana: Findings and implications
Source: PLoS One. 2024 Oct 29;19(10):e0313019. doi: 10.1371/journal.pone.0313019 (PMC11521307; doi:10.1371/journal.pone.0313019)
Supplement: Attachment — Submitted filename: RESPONSE TO REVIEWERS COMMENTS.docx [file pone.0313019.s001.docx]

**RESPONSE TO REVIEWERS COMMENTS**

Review Comments to the Author

Reviewer #1:

REVIEW COMMENTS: This study aims to investigate the level of appropriate use of antibiotics for diagnosed eye diseases at the ambulatory eye clinic of a teaching hospital. The study was conducted in good practice, supported by data and proper statistical analysis. The result showed that level of appropriateness is only 42% (!) and appropriateness of the choice of antibiotic was predicted only by patients’ age. This is quite shocked that author need to emphasize it and provide deeper analysis about this finding and its impact to the patients. It is also suggested to compare to other disease such as pneumonia, dental infections and urinary tract infections for broader perspective of antibiotics management. Please elaborate more in discussion section.

AUTHORS’ RESPONSE: Thank you for your comments. We have now added additional details in the discussion section on the finding of patient age being the only variable with significant association with appropriate choice of antibiotics. We have also added details of the comparison with other common infectious disease (pages 310-316).

Reviewer #2:

REVIEW COMMENTS: Review on topical antibiotics is limited. This data is important, even though this is a descriptive study, however the information is quite important. Some correlations somehow need to be clarify and confirmation such as how to explain the correlation between age with the prescription of antibiotics.

AUTHORS’ RESPONSE: Thank you for your comments. We have detailed information on the use of topical antibiotics and their advantage over systemic dosage forms in the discussion section on the use of topical antibiotics and its significance in the study (pages 324-332). We have added more details of the relevance of the finding of low appropriateness and its relationship with only age of the patients (pages 310-316)
